# The role of abdominal ultrasound in the management of excessive crying in infants

**DOI:** 10.11604/pamj.2018.30.68.12058

**Published:** 2018-05-28

**Authors:** Brahim El Hasbaoui, Lamia Karboubi, Badr Sououd Benjelloun

**Affiliations:** 1 Paediatric Medical Emergency Department, Children’s Hospital, Faculty of Medicine and Pharmacy, University Mohammed V, Rabat, Morocco

**Keywords:** Excessive crying, abdominal ultrasound, Intestinal intussusception, colic

## Abstract

Excessive or persistent crying is a common presentation to the pediatric emergency department, and often poses a diagnostic dilemma to emergency physicians. There are several reasons for excessive or persistent crying in children, ranging from benign causes like hunger, to life-threatening causes such as intussusception. The objective of this work is to specify the place of abdominal ultrasound in the diagnosis and management of incessant cries in the infant. A cross sectional investigation for 3 months about cases of infants admitted for excessive or persistent crying to the paediatric emergency medical department of the Rabat Children's Hospital. Thirty-nine cases of excessive crying. The average age of our patients was 5.7 months with a male predominance. The incessant cries constituted the main reason for consultation in all our patients. The abdominal ultrasound performed in all the patients and revealed six cases of "Intestinal intussusception, eight cases of colic with distention gas, one case of uretero-hydronephrosis, one case with lymphadenitis mesenteric whereas it was normal in twenty-three cases. Children presenting with excess or persistent crying with no clear historical and physical examination clues, pose a diagnostic challenge to emergency physicians. This survey illustrates that despite the fact that abdominal ultrasound was normal in 58% of the cases, it made possible to make an early diagnosis of 15% of acute intestinal intussusception and it has become the gold standard in management of excessive crying in infants.

## To the editors of the Pan African Medical Journal

Excessive crying is a fairly common presentation to the emergency department (ED) and needs comprehensive evaluation. In a classic study about crying in infants, Brazelton defines excessive crying as any amount of crying that worries the parents [1], but the consensus definition by several authors are the criteria defined by Wessel, known as the ‘‘rule of three’’ (crying spells at least three hours a day, three times a week for three consecutive weeks and lasting three months). Even with a consensus, there is no single definition of what should be considered excessive crying [2]. Most of the literature addresses excess crying in early infancy with focus on infantile colic. A large retrospective survey of infants presenting to the ED with excessive crying found serious underlying pathology in 5.1% of children, the most common cause identified is urinary tract infection [3]. Through this work we tried to determine the interest and role of abdominal ultrasound in the etiological research of incessant crying in infants.

we conducted a cross sectional investigation during three months about cases of infants admitted for excessive or persistent crying to the pediatric emergency medical department of the Rabat Children's Hospital, department that receives on average 150 cases per days of pediatric medical emergencies in its neonatal child and infant forms, incessant cries are the most common reason for consultation in infants under one year of age, we included in this prospective survey all infants from 3 to 12 months with incessant cries (crying spells at least three hours a day) with abdominal pain. Have been excluded incessant cries with extra abdominal diseases; reflux; otitis, trauma, bronchoalveolitis. We have collected data on the pregnancy, childbirth conditions, developmental milestones, the schedules of crying, triggering, calming factors and the associated signs. All our patients had a clinical examination and Ultrasound abdominal.

We have identified 39 cases of incessant crying, the average age was 5.7 months. Sex ratio: 1.78 in favor of boys. Their birth histories, their families’ social history, and developmental milestones were unremarkable. They were born at full term with no antenatal or perinatal complications. Prior to the symptoms, the children were described as a good eater, on a normal diet, and were thriving appropriately. Mothers reported crying/fussing: in 100% of cases, these cries were episodic in 51% of cases, repetitive in 18% of cases and continuous in about 13% of cases. The schedules of crying/fussing varied according to the circadian rhythm; 33% of the cases showed crying during the day; 20% of cases overnight and 28% of cases day and night. For all patients, there was no triggering factors for shouting, they could be calmed by parents in 31% of cases, by feeding in 10% of cases, by drugs (probiotics, antispasmodic) in 5% of cases, or spontaneously calmed in about 49% of cases (Table 1).

**Table 1:** Characteristics of the study population

**Number of cases**	39 cases
Average age	5,7 months
Sexe ration	1,78 (prédominance of males)
**Rhythm of crying**	
Episodic	51% (20 cases)
Repetitive	18% (7 cases)
Continuous	13% (5 cases)
Triggering factors	0
**Calming factors**	
By parents	31% (12 cases)
By feeding	10% (4 cases)
By drugs (probiotics, antispasmodic)	5% (2 cases)
spontaneously	49% (19 cases)

Excessive crying/fussing were associated with fever in 25% of cases; diarrhea 23% of cases; Constipation 2.5% of cases; Abdominal bloating 25% of cases; Abdominal pain 46% of cases; Sleep disorders 38% of cases (Table 2). Ultrasound abdominal was performed in all patients, it showed: Boudin of intussusception: 15% of cases; Large gas distortion: 20% of cases; Bilateral hydronephrosis: 2.5% of cases; Multiple mesenteric lymph nodes: 2.5% of cases; Ultrasound normal: 59% of cases

**Table 2:** Excessive crying associated factors

Factors	Number of cases	Percentage
Fever	10	25%
Diarrhea	9	23%
Abdominal pain	18	46%
Abdominal bloating	10	25%
Constipation	1	2,5%
Sleep disorders	15	38%

**Figure 1:**
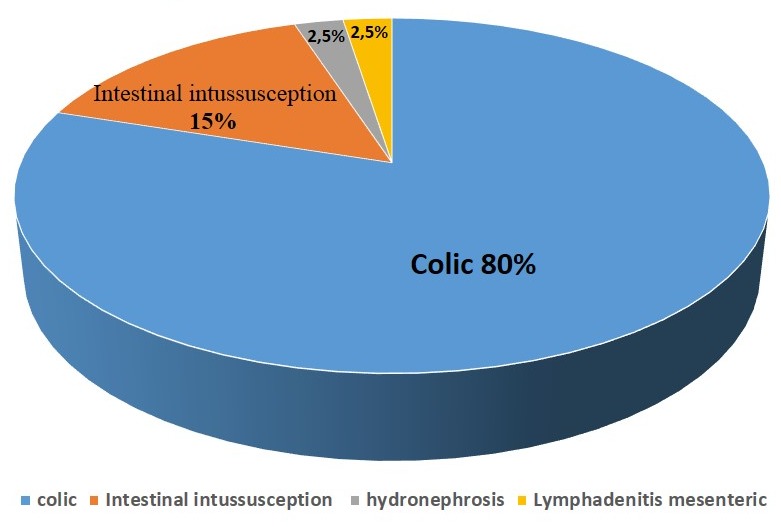
Causes of excessive crying in our serie

**Diagnosis retained** (Figure 1): Intestinal intussusception (15% of cases), colic (80% of cases), lymphadenitis mesenteric various pathologies: (2.5% of the cases); bilateral hydronephrosis: 2.5% of cases

Excessive crying is a common presentation to the ED and should be differentiated from transient crying which is frequently due to infantile colic [4]. Although there is no standard definition of excessive crying, it generally refers to crying that continues after caregivers have attempted to meet routine needs or crying that continues for longer than usual for a given child. While Wessel first described infant colic in 1954 [5], we still don’t know what causes it, or whether there is one cause or multiple. While the term “colic” implies an abdominal etiologic, there is little direct evidence for this localization. All that seems certain is that the babies are in distress. Wessel in fact seemed to recognize the uncertainty of colic’s underlying etiology and titled his manuscript, “Paroxysmal Fussing in Infancy, Sometimes Called Colic” [6]. It is important that we ultimately determine the cause of infant colic in order to manage these infants appropriately. Excessive and inconsolable crying can lead to caregiver frustration and can be a trigger for shaken baby syndrome, a form of child abuse with potential for significant neurologic morbidity [7]. Part of the reason many have assumed the etiology of infant colic is gastrointestinal is that the infants often pull up their legs and pass gas during the crying. There are several reasons for excessive or persistent crying in children. Common causes, are summarized in Table 3 [8]. Some of these causes can be excluded thorough history and a thorough physical examination.

**Table 3:** The most common causes of excessive crying in young infants

Colic	No apparent cause, healthy infant, gaining weight, rule of threes
Infections	Otitis media; urinary infection; meningitis
Gastrointestinal	Gastroesophageal reflus; reflus oesophagitis; constipation; intestinal intussusception; lactose intolerance or allergy to cow’s milk
Trauma	Corneal abrasion; foreign body in the eye; toe-tourniquet syndrome (strangulation of digits)
Behavioral/interactional	Excessive stimulation, lack of routine, bonding disorder
Drug reactions	Reactions to vaccins; drugs used during pregnancy (narcotics)
Violence/abuse	Long bone fractures; eye hemorrhage; intracranial haemorrhage
Hematological/cardiovascular	Hemolytic crisis, sickle cell anemia, tachyarrhythmie, congestive heart failure

The Intussusception is the most common abdominal emergency in infancy and early childhood, but it can occur at any age. It’s defined by penetration of an intestinal segment into the distal contiguous segment causes obstruction of the digestive lumen and vascular compression at the neck level. It occurs between 2 months and 2 years with a frequency peak between 6 and 9 months. This is the case of our patients who are in this same age group. In most cases (> 95%), the intussusception is called "idiopathic" [9]. The traditional triad of revelatory signs paroxysmal abdominal pain, vomiting and rectal bleeding. It is rarely complete, currently in industrialized countries [10]. Abdominal ultrasound has become the gold standard to diagnose or exclude intussusception [10]. It enables identification of the anatomic form, suspicion of vascular compromise, and depiction of a focal pathological lead point, providing guidance for therapeutic management. In our study the intestinal intussusception was found in 6 cases, they had between 5 and 11 months, the circumstances of the diagnosis were abdominal pain and bloating, their crying was repetitive, abdominal ultrasound found a bullion of intussusception, and therefore early surgical management. On the other side cow’s milk protein allergy may play a causative role in a proportion of formula-fed colicky infants, their symptomatology may distinguish them from those with idiopathic infant colic. Indicators of dietary protein hypersensitivity and intestinal damage, such as alpha-1 antitrypsin and fecal hemoglobin, are not elevated in babies with infant colic [11].

There does not seem to be evidence that colicky babies are suffering from lactose intolerance. While supplementation with probiotics appeared promising in one group’s experience, their benefits have not been reproduced. In addition idiopathic colic is characterized by the occurrence of intense cries usually at the end of the day, soon after the meal, while the baby begins to fall asleep. Motor agitation and crying lead to acute abdominal pain related to spasmodic attacks of the terminal intestine. The use of probiotics is part of several studies and is increasing as a promise of improvement in colic symptoms, but the evidence of its effectiveness and the results are controversial. In a recent meta-analysis there was an improvement in crying and treatment effectiveness, but only after two to three weeks and together with the natural history of colic improvement. In another systematic review and meta-analysis study, the improvement occurred only in the group of infants that was breastfed and the study concluded that there is little evidence to recommend the use of probiotics in the treatment of infants with colic [12]. In our cases colic was responsible for 80% of excessive infant crying, they had 3 at 4 months and presenting episodic crying. Furthermore Hydronephrosis or Pyelocalcic dilatation (PC) is the result of a functional alteration of the mechanism of transport of the urine of the pelvis to the ureter; the embryological origin of the observed histological abnormalities remains unknown. The incidence of congenital hydronephrosis is about 5/100 000. It is more frequent in men than in women (ratio of 2/1 to 5/2), usually left (ratio 5/2) and bilateral in 10 to 15% of cases. Intermittent pains in the abdomen, flank or lumbar fossa, associated or not with nausea or vomiting, are the most frequent signs of pyelocalcic dilatation.

## Conclusion

The crying infant represents a difficult diagnosis dilemma and may be the primary manifestation of a serious or even life-threatening condition. The emergency physician can effectively evaluate the child through comprehensive history, physical examination, and ancillary resources to rule out potentially life threatening processes. The clinician must also effectively manage the anxious and frustrated caregiver who presents with the child. The primary care provider is an important resource in the management of this very stressful problem.

## Competing interests

The authors declare no competing interests.
